# Comparison and selection of patient follow-up systems for covid-19 pandemic patients

**DOI:** 10.1186/s40691-022-00296-7

**Published:** 2022-08-05

**Authors:** Tamer Eren, Tuğba Danışan, Ayşegül Deringöz, Güler Aksüt

**Affiliations:** 1 Department of Industrial Engineering, Kırıkkale University, Kırıkkale, 71450 Turkey; 2 Doctor of Occupational Health and Safety, Ministry of Education, Yozgat, 66100 Turkey

**Keywords:** Wearable technology, Remote patient monitoring systems, Covid-19, Multi-criteria decision making (MCDM), Selection

## Abstract

People have struggled with many infectious diseases throughout history. Today, the Covid-19 is being fought. One of the most important things for people who have or are at risk of getting Covid-19 is social isolation. Many countries resort to different ways to ensure social isolation. For this, remote patient monitoring systems have been developed. In this study, the problem of the selection of Covid-19 remote patient monitoring systems is discussed. Seven Wearable Health Technology (WHT) products were evaluated with a total of 10 criteria, including the important symptoms used in the patient tracking systems. The weights of 10 criteria determined by the Analytical Hierarchy Process (AHP) method were calculated, and these weights were used in the solution of The Preference Ranking Organization Method for Enrichment Evaluation (PROMETHEE), and Technique for Order Preference by Similarity to Ideal Solutions (TOPSIS) methods. WHT products were compared. As a result, the most appropriate patient follow-up system was determined. This study generates differences in terms of evaluating seven different products and ten criteria in total with MCDM methods. A more comprehensive evaluation has been made in the literature than the studies in this field.

## Introduction

Contagious epidemics, in other words, epidemics, are contagious diseases that spread to vast areas, sometimes to a continent or even to the whole world, causing illness and death in humans or animals. All infectious diseases are capable of transmitting to humans or animals in various ways (Aslan, 2020). Covid-19 is a large family of viruses that cause disease in humans and animals (Dikmen et al., 2020). Covid-19, which emerged in China towards the end of 2019, affected more than 170 countries in 4.5 months (Ankaralı et al., 2020). The Covid-19, also known as Covid-19, poses a great danger to humanity.

With the development of technology, wireless communication has spread to all areas (Yılmaz & Güven, 2017). For remote patient monitoring, systems are placed directly on the patient, and measurements of the patient can be made via the placed device (Groff & Mulvaney, 2000). Patient tracking systems developed for Covid-19 patients serve to keep the spread under control for patients who are infected or at risk of being infected and to monitor the health status of infected people remotely. The use of these systems increases the distance between the doctor and the patient and reduces the risk of infection of doctors and all health workers. At the same time, following the infected person by staying at home helps to reduce the occupancy rate of hospitals. One of the most effective features in the spread of the epidemic is contact. Increasing social distance and reducing contact with people is the best method to prevent the epidemic. However, isolation is very important for people who are infected and at risk of contracting the epidemic (URL 1). Designed for this, Wearable Health Technology (WHT) helps in remote patient monitoring. In the study, WHT products that will enable remote follow-up for Covid-19 patients were evaluated. In the solution process, Analytical Hierarchy Process (AHP), Preference Ranking Organization Method for Enrichment Evaluation (PROMETHEE), and Technique for Order Preference by Similarity to Ideal Solutions (TOPSIS) methods, which are easily applicable and frequently used in selection problems in the literature, were used. As far as is known, this study is different from other studies in the literature;

- In the evaluation of WHT products, the criteria are temperature monitoring of the body, respiratory rate monitoring, the weight of the product, whether it is water-resistant, sleep tracking, activity tracking, distance tracking, battery life of the product, price, and whether it is single or multi-use.
- It creates differences in terms of evaluating seven different products in total with Multi-Criteria Decision Making (MCDM) methods, with four previously evaluated products and three newly added products.
- A more comprehensive evaluation has been made in the literature than the studies in this field.

In the second part of the study, the literature on the subject is presented. In the third chapter, the method followed in the study and the solution steps is given. Finally, the results of the study are discussed in the fourth section.

## Literature review

Although more than one study has been conducted in the literature on wearable devices, treatment tracking systems, and telemedicine for Covid-19, Lakkireddy et al. (2020) conducted a study describing the strategies for patients affected by Covid19 and the role of remote telemedicine. Gorodeski et al. (2020), while describing the importance of virtual care for people with chronic conditions, Sun et al. (2020), aiming to limit the spread of Covid-19 through the mobile health platform, investigated the benefits of applications that monitor patients with the help of wearable devices. Zhu et al. (2020) used the heart rate and sleep data collected from wearable devices in people living in different countries and cities to describe the epidemic trend of Covid-19 with the prediction model they developed. Wosik et al. (2020) examined how people, processes, and technology work and can work together for a successful telehealth transformation in the Covid-19. Singh et al. (2020) described the solution that controls the spread of the disease by developing an IoT-based quarantine tape for monitoring people with a positive diagnosis of Covid-19. Tavakoli et al. (2020) explained the benefits of smart wearable devices to healthcare and how they support healthcare personnel during the Covid-19. Zhang et al. (2020) remotely monitor the health status of the medical aid team working at Wuhan Huoshenshan Hospital, showing how sensitive monitoring is effective in increasing work efficiency and sustaining the workforce in emergency situations such as pandemics of healthcare workers around the world. Alwashmi (2020) has explored the application potential of digital technologies that can be used at different stages of the Covid-19 outbreak, including data-driven disease surveillance, screening, diagnosis, and monitoring. Ktori (2020) evaluated the data of a disposable wearable sensor that collects physiological data of patients with heart failure and showed that it could be predicted whether the patient will be hospitalized ten days in advance. Öcal et al. (2019) evaluated the internet of things in smart and traditional wearable health devices. These studies are generally studies on disease definition, diagnosis, and treatment. İmren (2011) researched the optimal business location problem in the furniture industry and solved it with AHP. Özkan (2007) examined the personnel selection process of an enterprise using MCDM (AHP, TOPSIS, ELECTRE) methods. Cihan et al. (2016) solved the planned echocardiography device selection problem with AHP and TOPSIS methods. Aydın and Eren (2018) solved the problem of choosing the best supplier for the defense industry with AHP and TOPSIS methods. Vaidya and Kumar (2006) presented a literature review of AHP applications. Zebrardast (2002) demonstrated the application of AHP for urban and regional site selection purposes. Gür et al. (2016) made the selection of the most suitable transportation projects for the route determined in Ankara Metropolitan Municipality. Bedir and Eren (2015) solved a sales consultant selection problem of a company. Eren and Özder (2016) selected the supplier company for the necessary material in the production process of the beverage company. Eren et al. (2018) evaluated cardiovascular surgery polyclinics with multi-criteria decision-making methods. Liberatore and Nydick (2008) conducted a literature review of the applications of the AHP method in medicine and health. Alağaş et al. (2017) aim to determine the most efficient advertising strategy for a furniture company operating throughout Turkey by optimizing the budget they allocate for advertising expenditures. Asaoğlu and Eren (2018) A company has chosen a cargo company.

At the same time, studies have been carried out for wearable technologies in the literature. Turgut et al., (2020, 2021), Deringöz et al., (2021a, 2021b), Akıncı et al., (2021a, 2021b) conducted studies. From these studies, Turgut et al. (2020) evaluated smartwatches using the activity tracking criterion used for those who do sports. Deringöz et al. (2021a) selected wearable technologies for Covid 19 patient follow-up, taking into account the blood pressure criteria used in the follow-up of hypertension patients. Akıncı et al. (2021a) carried out studies that consider the examination and selection of wearable technologies for obese patients. Along with this study, Akıncı et al. (2021b) evaluated these technologies for hypertension patients. Apart from the healthcare field, Deringöz et al. (2021b) evaluated industrial wearable technologies. For the first time in the application areas of these studies, wearable technologies were evaluated with multi-criteria decision-making methods.

As far as is known, the study is the first in terms of scope to evaluate WHT products in the literature. Body temperature tracking, respiratory rate tracking, product weight, water resistance, sleep tracking, activity tracking, distance tracking, product battery life, price, single-multi-use criteria are used. Seven different alternatives were evaluated.

## Methods

**Fig. 1:**
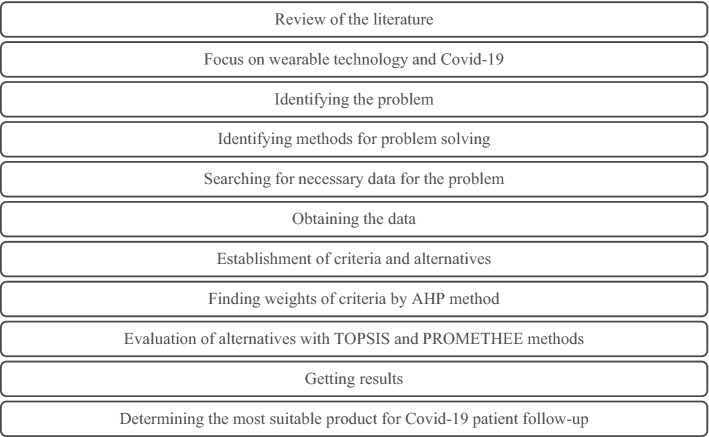
Implementation steps. *AHP* Analytical Hierarchy Process, *TOPSIS* Technique for Order Preference by Similarity to Ideal Solutions (TOPSIS), *PROMETHEE* Preference Ranking Organization Method for Enrichment Evaluation

In this study, unlike the studies in the literature, WHT products designed to be used in the patient tracking systems were compared. First of all, six criteria, including body temperature, amount of oxygen in the blood, being in contact with patients or carriers (URL 2), which are effective in Covid-19 patient follow-up, were determined. The weights of these criteria were found by the AHP method, and these weights were used in the PROMETHEE and TOPSIS methods where WHT selection was made. While working, the steps in Fig. 1 were followed.

### AHP method

**Fig. 2:**
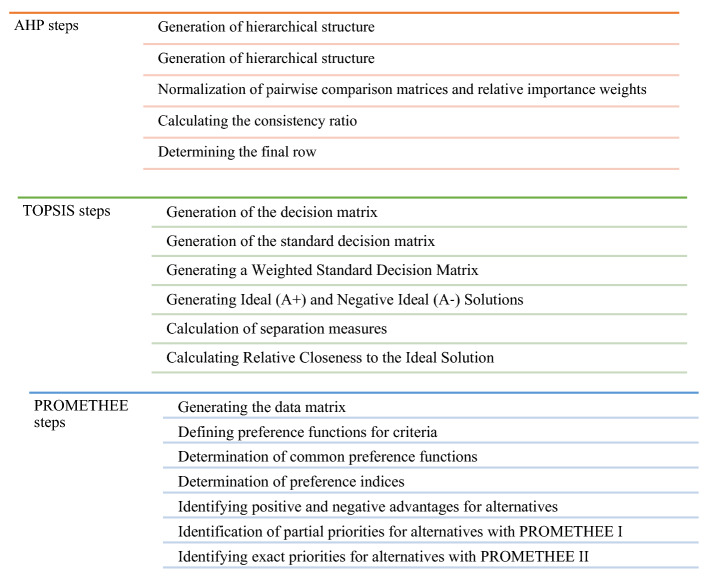
The steps of the Analytical Hierarchy Process (AHP), Technique for Order Preference by Similarity to Ideal Solutions (TOPSIS) and Preference Ranking Organization Method for Enrichment Evaluation (PROMETHEE) methods

The AHP method allows the evaluation of more than one criterion. AHP is an effective tool for complex decision-making problems (Aydın & Eren, 2018). With the AHP method, complex contents are simply expressed with a hierarchical structure and examined with intuitive and logical thinking (Toksarı & Toksarı, 2011). The steps of the AHP method are as follows in Fig. 2 (Özcan, Ökten, et al., 2020a; Saaty, 1980):

### TOPSIS method

The TOPSIS method emerged with the shortest distance to the positive-ideal solution and the longest distance approach to the negative-ideal solution of the alternatives (Hwang & Yoon, 1981; Özden, 2015). It consists of six steps in Fig. 2.

### PROMETHEE method

It is a method developed by Brans et al. in 1982 (Bedir & Eren, 2015). It consists of two stages, Promethee 1 (partial sorting) and Promethee 2 (full sorting). PROMETHEE method consists of seven steps in Fig. 2 (Brans, 1982).

### Application

One of the most important factors for people who have or are at risk of contracting Covid-19 is the provision of social isolation (URL 1). Many countries resort to different ways to ensure social isolation. Many solutions have been developed for social isolation. Since the increase in social isolation reduces the spread of the disease, various patient follow-up technologies and systems are an effective method both in increasing isolation and reducing the occupancy rates in hospitals. It also protects hospital workers against contamination (URL 2). In this study, the problem of choosing the most suitable product from WHT products is discussed in order to remotely monitor people who have Covid-19 or work in a business and to detect those who are sick and at risk of disease early. While the AHP method was used to calculate the weights of the criteria in the problem, these weights were used in the solution of the PROMETHEE and TOPSIS methods for the selection of the products, and the solution was made.

#### Covid-19 patient tracking systems

Covid-19 is an infectious disease that affects people's lives, caused by the severe acute respiratory syndrome SARS-CoV-2. Despite the fact that the symptoms seen in the cases are similar, some of the patients have mild symptoms, but some of them also lead to severe diseases such as pneumonia and multi-organ failure (URL 2). The use of WHT products for remote monitoring of patients who are infected or at risk of being infected will facilitate the isolation of people. Thus, the distance between patients and healthcare professionals will increase, and the occupancy rate in hospitals will decrease. For these reasons, more than one country has developed various solutions for remote patient follow-up. Countries are planning to reduce the spread and contagiousness to a minimum level by remote patient follow-up with the GST products they have developed (URL 3; URL 4). Covid-19 patient tracking systems and features: It has developed a continuous monitoring and alerting solution aimed at automating continuous monitoring of changes in patient temperature with VivaLNK (URL 5). LifeSignal, a disposable patch for early detection and monitoring of Covid-19, is simply attached to the chest area and records data in real-time (URL 6; URL 7). Loop signal assists in-home monitoring of patients confirmed, suspected, or at risk for Covid-19 (URL 8). The Bio Button, on the other hand, measures 90 days of continuous temperature and other vital signs in-home patients, high-risk individuals, and frontline healthcare professionals. It is a coin-sized, disposable medical device (URL 4; URL 9) that allows them to return to work and school safely. Vital Patch Biosensor is a health monitoring device. State-of-the-art biosensor continuously monitors eight physiological parameters in real-time (URL 10). The Oura Ring wearable fitness and health tracker is a convenient way for people to track their activity level and physical response and support their personal fitness goals (URL 11). Biostrap takes a high-resolution snapshot of biometric data such as heart rate, heart rate variability, oxygen saturation, and respiratory rate (URL 12). Table 1 includes WHT products.

**Table 1:** WHT products and features used in Covid-19 tracking

	VivaL NK	Life signals	Loop signal	Bio button	Vitalpatch biosensor	Oura ring	Biostrap
Heart rate tracking	*	*	*	*	*	*	*
Abnormal heart rhythm warning					*	*	*
Body temperature monitoring	*	*		*	*	*	
Distance tracking		*		*			
Measurement of the amount of oxygen in the blood		*	*	*	*	*	*
Calorie tracking		*		*			
Water resistance	*		*			*	*
Activity tracking		*		*	*	*	*
Real-time data transmission	*	*	*	*	*	*	
Respiratory rate monitoring	*	*	*	*	*		*
Sleep tracking				*		*	*
Make a call or send a message			*				
Cough tracking				*			
Vomiting follow-up				*			
Contact tracing			*	*			
Sneeze tracking				*			
Price	32 $	55 $	90 $	38 $	41 $	249 $	299 $
Battery life	168 h	120 h	72 h	2160 h	168 h	120 h	60 h
Weight	7.5 g	2 g	18 g	2.5 g	13 g	4 g	80 g
Single or reusable	Very	Very	Very	Very	Single	Very	Very

#### Determination of criteria

Covid-19 is a disease in which different symptoms are observed in people. According to the information in the Covid-19 patient treatment guide (URL 1) published by the Ministry of Health of the Republic of Turkey, there are some vital signs that should be monitored first. It is obligatory to monitor these findings, and as a result of this monitoring, various follow-up and treatment processes such as self-quarantine in the hospital or at home are operated on depending on the patient's condition. Especially the risk of developing the severe disease is higher in the second week of the disease. Real-time monitoring and Patient tracking systems is of critical importance since it is important to continue their treatment in the hospital in cases such as shortness of breath and non-reduction of fever (URL 1);

- Heart rate and rhythm
- Respiration rate
- Blood pressure
- Body temperature
- The amount of oxygen in the blood (URL 1).

The criteria chosen in this direction and their explanations are given in Table 2.

**Table 2:** Criteria and explanations

Criteria	Explanation
Respiration rate (1)	It is the criterion that shows whether the product has a respiratory rate tracking feature
Body temperature tracker (2)	It is the criterion that shows whether it has the feature of measuring body temperature
Disposable or reusable (3)	It is the criterion that indicates whether the product is single or multi-use
Battery life (4)	It is the criterion that shows how many hours the battery can be used after charging the product or if it is disposable
Price (5)	It is the criterion that indicates the selling cost of the product
Weight (6)	It is the criterion that explains how many grams the product weighs
Water resistance (7)	It is the criterion that shows whether the product is water resistant
Sleep tracker (8)	It is the criterion that indicates whether the product monitors sleep or not
Activity tracking (9)	It is the criterion that indicates whether the product is tracking activity
Distance tracking (10)	It is the criterion that indicates whether the product can track distance or not

#### Determination of criterion priorities with AHP method

**Fig. 3:**
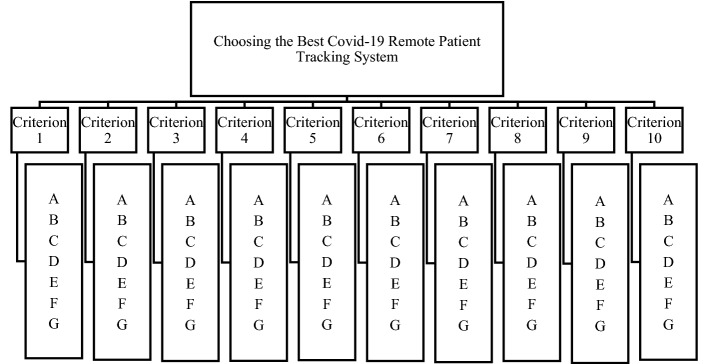
Hierarchical structure for the Covid-19 patient tracking system selection problem

First, the purpose is determined, then the criteria that affect the selection in line with the purpose are put forward. In line with the purpose of the decision-maker, criteria and their sub-criteria are determined, and a hierarchical structure is created. The hierarchical structure created for Covid-19 remote patient monitoring systems is given in Fig. 3.

On the basis of the criterion, a pairwise comparison matrix was created, and the priority values of each criterion were determined. Pairwise comparison matrix and significance values are shown in Table 3. As a result of the AHP method, the consistency of the matrix created on the basis of the criterion was found to be 0.099. It is concluded that the comparison matrix formed is consistent. According to the results obtained, the first priority criterion is the respiratory rate monitoring criterion. Ministry of Health of Turkey reports were also considered (URL1, 2020). Evaluations were made by three academicians and three public health experts for evaluation in the study. In addition, in determining the criteria.

**Table 3:** Pairwise comparison matrix and significance values

Criteria	1	2	3	4	5	6	7	8	9	10	Importance values
Price (1)	1.00	0.50	0.50	0.33	3.00	0.33	0.33	2.00	2.00	2.00	0.085
Battery life (2)	2.00	1.00	2.00	0.33	2.00	0.33	0.33	2.00	2.00	2.00	0.095
Weight (3)	2.00	0.50	1.00	0.33	0.50	0.20	0.20	0.50	0.50	0.50	0.044
Disposable or reusable (4)	3.00	3.00	3.00	1.00	2.00	0.33	0.33	0.50	0.50	0.50	0.097
Water resistance (5)	0.33	0.50	2.00	0.50	1.00	0.33	0.33	2.00	2.00	2.00	0.076
Body temperature tracking (6)	3.00	3.00	5.00	3.00	3.00	1.00	0.50	3.00	3.00	3.00	0.189
Respiratory rate monitoring (7)	3.00	3.00	5.00	3.00	3.00	2.00	1.00	3.00	3.00	3.00	0.219
Sleep tracking (8)	0.50	0.50	2.00	2.00	0.50	0.33	0.33	1.00	1.00	1.00	0.065
Activity tracking (9)	0.50	0.50	2.00	2.00	0.50	0.33	0.33	1.00	1.00	1.00	0.065
Distance tracking (10)	0.50	0.50	2.00	2.00	0.50	0.33	0.33	1.00	1.00	1.00	0.065

#### PROMETHEE solution

**Fig. 4:**
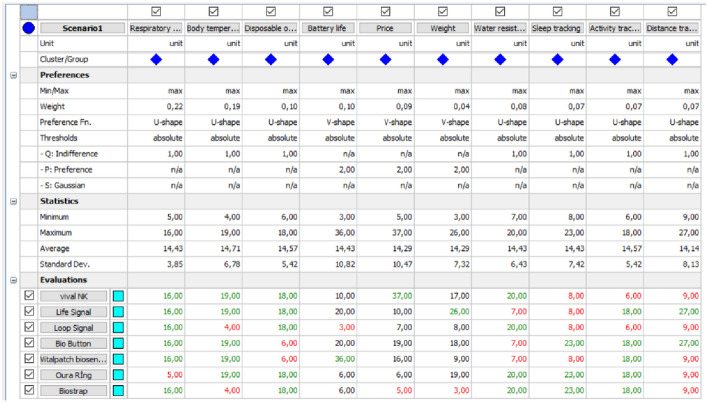
Preference Ranking Organization Method for Enrichment Evaluation (PROMETHEE) program interface

At this stage of the study, criterion weights obtained by the AHP method were used. The criteria taken into account in the evaluation of alternative products, the values determined on the basis of the Criteria, and the weights assigned to each criterion were entered into the Visual PROMETHEE (URL 13) package program, and the results were obtained. The page where the decision matrix is entered is given in Fig. 4.

**Fig. 5:**
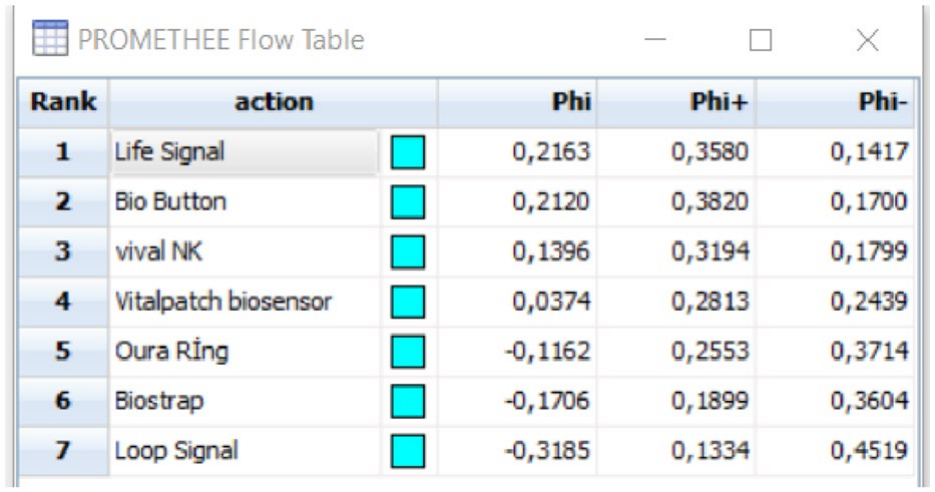
Preference Ranking Organization Method for Enrichment Evaluation (PROMETHEE) solution result

Each valuation measure is a true value (qualitative measures expressed in numerical values). Change functions are determined for each criterion. In the study, the U type function was used for the criteria with 0 and 1 values, and the V type function was used for the criteria with numerical values. The values obtained as a result of the solution are given in Fig. 5.

As a result of the method, it was concluded that the product that should be selected is the Life Signal product.

#### TOPSIS solution

At this stage of the study, the matrix in Table 4 was used for the TOPSIS method. In the last column of the same table, the priority values obtained as a result of the method are also included.

**Table 4:** The decision matrix used in the TOPSIS method and the solution result

	A	B	C	D	E	F	G	H	I	J	Priority value
VivaL NK	16	19	18	10	37	17	20	8	6	9	0.60
Life signals	16	19	18	20	10	26	7	8	18	27	0.57
Loop signal	16	4	18	3	7	8	20	8	6	9	0.38
Bio button	16	19	6	20	19	18	7	23	18	27	0.52
Vitalpatch biosensor	16	19	6	36	16	9	7	8	18	9	0.43
Oura ring	5	19	18	6	6	19	20	23	18	9	0.54
Biostrap	16	4	18	6	5	3	20	23	18	9	0.37

As a result of the TOPSIS method solution, the VivaL NK product was the product that took the first place, and it was concluded that it should be selected. As a result of the problem solutions, priority values for WHT products were calculated. The obtained solution results are compared in Table 5.

**Table 5:** Ranking of WHT products by methods

Ranking	TOPSIS	PROMETHEE
1	VivaL NK	Life signal
2	Life signal	Bio button
3	Oura ring	VivaL NK
4	Bio button	Vİtalpatch biosensor
5	Vİtalpatch biosensor	Oura ring
6	Loop signal	Biostrap
7	Biostrap	Loop signal

As a result of the solutions, the Life Signal product, which is in the first place in the PROMETHEE method, is in the second place in the TOPSIS method. VivaL NK product, which took first place in the TOPSIS method, took third place in the PROMETHEE method. As a result of the comparison of the solutions, it was concluded that the choice of Life Signal product would be more accurate.

## Discussion

WHT products, developed to reduce the contagiousness of the Covid-19, minimize doctor-patient contact, and reduce the occupancy rates in hospitals, make a great contribution to preventing the spread of the disease by remote patient follow-up. In this study, 7 WHT products that are currently approved and used in various institutions have been taken into account in order to patient tracking systems today and to keep track of both patients, those at risk, and employees in a business or institution.

In the evaluation of the products, the guide published by the Republic of Turkey Ministry of Health, General Directorate of Public Health (URL 1), and the report published by the World Health Organization (URL 2) were used. Evaluations were made by three academicians and two public health experts. Oxygen amount in the blood, body temperature and contact tracing are the criteria that should be followed primarily in the diagnosis and treatment of Covid-19. In addition, it was determined that the price of the product, battery life, single or multi-use criteria should be taken into account. In the solution phase, first of all, three academicians and two public health experts made evaluations, and the necessary information for MCDM methods was determined. In the first stage of the study, criterion weights were obtained using the AHP method (Özcan et al., 2019), which is frequently used in the literature and provides effective results. After the criterion weights are found, the PROMETHEE method is preferred because there are criteria that take 0 and 1 values in the problem structure, and there are various functions in it to solve this structure appropriately. Initial evaluation has been made for WHT product selection. In order to compare the results obtained with these methods, a solution has also been made with the TOPSIS method (Özcan, Danışan, et al., 2020), which has an easy solution process among the ranking algorithms in the literature and provides effective results.

The product priorities obtained as a result of these two methods were compared, and it was concluded that the most suitable product to be used in Covid-19 tracking was the Life Signal product. As far as the study is known, in the evaluation of WHT products in the literature; It is the first in terms of body temperature tracking, respiratory rate tracking, the weight of the product, whether it is water-resistant, sleeps tracking, activity tracking, distance tracking, battery life of the product, price and whether it is single or multi-use. In this respect, Deringöz et al. (2021a) are different from the study. It is more comprehensive. Seven alternatives were evaluated within the framework of ten criteria.

Current data were used for the products specified in the study. But in the future, more advanced products may have different features. In addition, new criteria can be added inpatient follow-up according to Covid-19 mutations. It constitutes the limits of this study. The methodology proposed in this study can be followed in future studies and effective results can be obtained. Only criteria and alternatives can be updated. And in future studies in this area, other health technologies developed with WHT products being developed for Covid-19, as well as the difficulties in implementing products or systems to be used for Covid-19, can be evaluated in this process.

## Data Availability

Not applicable.
